# Online oral health information seeking experience and knowledge, attitudes and practices of oral health among iranian medical students: an online survey

**DOI:** 10.1186/s12903-022-02061-0

**Published:** 2022-02-04

**Authors:** Peivand Bastani, Mohammad Amin Bahrami, Kostas Kapellas, Alireza Yusefi, Giampiero Rossi-Fedele

**Affiliations:** 1 Faculty of Health and Behavioral Sciences, School of Dentistry, University of Queensland, Brisbane, QLD 4072 Australia; 2 School of Health Management and Information Sciences, Health Human Resource Research Center, Shiraz University of Medical Sciences, Shiraz, Iran; 3 Australian Research Centre for Population Oral Health, Adelaide Dental School, Adelaide, Australia; 4 Department of Public Health, School of Health, Jiroft University of Medical Sciences, Jiroft, Iran; 5 Adelaide Dental School, Adelaide, Australia

**Keywords:** Internet, Oral health, Information literacy, Information seeking behavior, Knowledge, Attitude, Practice

## Abstract

**Background:**

The internet is increasingly used as a source of health information. This study aimed to explore the online oral health information seeking experience, to determine the knowledge, attitudes and practices (KAP) of oral health, and to investigate the associations between online oral health information seeking experience and oral health KAP of participants.

**Methods:**

A cross-sectional online survey was conducted. Three hundred and ninety-five university students participated in the study. Required data were gathered using two valid questionnaires eHIQ (e-Health Information Questionnaire) and Oral Health Knowledge, Attitude and Behavior Questionnaire. eHIQ was a 2-part instrument with 37 items. eHIQ-Part 1 includes 11 items related to general views of using the internet in relation to health. eHIQ-Part 2 includes 26 items related to the consequences of using specific health-related online sources. The second questionnaire includes 30 items as a combination of multiple-choice and yes/no type questions. The data were analyzed using the statistical analysis software SPSS version 20. Mean scores, standard deviation, and frequency distribution were obtained. Independent T-test, correlation coefficients and analysis of variance (ANOVA) were used in the analysis.

**Results:**

Participants had good KAP of oral health. The between-group differences tests showed that oral health knowledge and attitudes were significantly different between gender and years of study groups, but the differences of oral health practices were significant only based on years of study. Participants had moderate scores regarding all sub-scales of eHIQ-Parts 1 and 2. Findings revealed that online oral health information seeking behavior was associated with oral health KAP (*p* < 0.05).

**Conclusion:**

According to the results the general views of using the internet in relation to health and the consequences of using specific health-related online sources were in a moderate level among the participants. Such results can emphasize the need for more planning, education and empowerment of the population`s health literacy. The present study also provides good insights for the latter and has practical and policy implications besides its research values.

## Introduction

Oral health is considered a determinant indicator for the community’s general health and wellbeing [1]. Many factors can widely affect the oral health status of the population, among them include socioeconomic factors [2] and health behaviors [3]. The latter is mainly focused on preventive interventions and behaviors and is determined by the level of information and literacy of the community, particularly about the causal factors of dental caries and the effective modes of prevention [4].

Health information seeking behavior is considered as any action by individuals through which they want to increase their knowledge or information about a health issue in order to improve their health [5]. People`s attitudes and knowledge can incrementally affect their health seeking behaviors. According to Andersen’s health behavior model, some individuals` health condition and their perceived consequence of the action or inaction can determine the health seeking behaviors [6].

Many interventions can be applied to improve the oral health seeking behaviors such as: developing the target population`s education and awareness particularly on applying preventive strategies, implementing and developing health financing interventions and insurance packages and creating opportunities for increasing the access of the population for appropriate time and place of seeking oral health caries treatments [7].

Considering the relevant literature, online health services are among one of the ways to increase access and potentiality of seeking oral health behaviors. In this regard, Hanna et al. (2017) emphasized that, although oral and dental health care professionals are defined as the main trusted and reliable sources for seeking information, other sources are commonly used by patients such as online health services [8]. The degree of effectiveness and usefulness of such online information seeking is highly dependent on the health and internet literacy of the population in addition to access to information and communication technology (ICT) [9].

Health seeking behaviors intend to improve ones’ health, and in the process, that of the community. Developing online health resources? are necessary, in particular for youth and those in the most literate parts of the population such as the students. Although medical students are among those groups with an appropriate health literacy level and acceptable access to internet for information seeking, it is not clear whether they have a high level of health information seeking behavior or not particularly in a developing country. In another words, the question is that if the medical students appropriate access to internet will lead to an acceptable level of health information seeking behavior or not? In this regard, results of Islam et al. (2017) in Bangladesh have shown that the university students` seeking via the internet is not satisfactory and there is a serious necessity for enhancing the applied programs and improving the incentives including appropriate training and empowering them in seeking, interpreting and evaluating the medical and health related information [10].

To fill the present gaps and determine the status of online health seeking behavior in the area of oral and dental health, the present study is aimed to determine the attitude of Iranian medical students towards online oral health information seeking and their level of knowledge, attitudes and oral health practices in this area. The present results can inform policymakers to apply more appropriate and applicable plans and interventions to improve the oral health seeking behaviors among the community particularly in the developing contexts with the similar setting.

## Methods

### Design and objective

An online cross-sectional questionnaire survey was carried out in 2020–2021 in Iran. This study was started in Nov 2020 to April 2021 in a duration of about 6 months. The aims of the study were: (1) determine the attitudes of participants toward online oral health information and sharing information online; (2) determine the level of knowledge, attitudes and oral health practices of participants; (3) determine the correlation of oral online health information seeking experience of participants with their knowledge, attitudes and oral health practices.

### Participants

The study population included Shiraz University of medical sciences students who were studied in an undergraduate discipline. For more heterogeneity of the study population, all the medical and para-medical majors in the BSc degree were included. The medical students were included before their internship period for decreasing the impact of more clinical knowledge and increasing the possibility of comparison among different groups. Those who did not have access to the internet for search or did not search oral health information in the past three months were excluded from the study. The link of an online survey was distributed through the social media groups in WhatsApp which was originally created for virtual education during COVID-19 pandemic. Participants were fully debriefed about the objective of the survey. For this purpose, right after opening the shared link, they had to read a brief description about the aims of the study and voluntary anonymous nature of the participation and check the pre-determined box, then they were allowed to enter to the next part and answer the questions including the demographic variables and the study questionnaires.

### Ethical considerations

All participants provided an online informed consent to participate in the study and were assured that their information would be kept confidential. All study procedures were conducted in accordance with the ethical standards of the Declaration of Helsinki. This study was approved by the ethics committee affiliated with Shiraz University of Medical Sciences with the ID of IR.SUMS.REC.1397.373.

### Instruments

The online survey included demographic variables and two self-reported structured questionnaires:

1. eHIQ (e-Health Information Questionnaire part 1 and 2) [11]: eHIQ was used to measure the online health information seeking behavior of participants. The eHIQ, developed by Kelly et al. in 2015 as an instrument to measure the potential consequences of using websites containing different types of material across a range of health conditions, is a 2-part instrument with 37 items. eHIQ-Part 1 includes 11 items related to general views of using the internet in relation to health. 11 items of eHIQ-Part 1 have been grouped into two sub-scales named Attitudes towards online health information (5 items) and Attitudes towards sharing health experiences online (six items). Via these 11 questions the participants were asked to determine to what extent they agreed or disagreed with the reliability, helpfulness, usefulness and assuredness of internet for health information seeking.
  eHIQ-Part 2 includes 26 items related to the consequences of using specific health-related online sources. 26 items of eHIQ-Part 2 also have been grouped into three sub-scales including Confidence and identification (nine items); Information and presentation (eight items) and Understanding and motivation (nine items). These questions were asked to show the participants` agreement with the face, content and the helpfulness and robustness of the health websites they were used for information seeking. It should be mentioned that in our study, the participants were asked to respond to the 26 items of eHIQ-Part 2 regarding the online sources which they have sought during the recent months. Also, the participants were asked to score all items of both parts on a 5-point scale ranging from ‘never’ to ‘always' scored 1–5. The mean scores between 1–2.33, 2.34–3.66 and higher than 3.66 were defined as low, moderate and high scores.
  We used a standard ‘forward–backward’ procedure to translate the eHIQ from English into Persian. To demonstrate the content validity, we used the content validity ratio to quantify the extent of the experts’ agreement. The reliability of the translated version of the eHIQ was confirmed using Cronbach’s alpha coefficient that was calculated as 0.89 for the total scale and 0.81, 0.87, 0.94, 0.83 and 0.91 for Attitudes towards online health information, Attitudes towards sharing health experiences online, Confidence and identification, Information and presentation, and Understanding and motivation, respectively.
2. Oral Health Knowledge, Attitude and Behavior Questionnaire [12]: To determine oral health knowledge, attitudes and practices of participants we used a relevant questionnaire developed by Ahmed et al. (2015). The questionnaire includes 30 items in three sections for the evaluation of oral health knowledge, attitudes and behaviors of the respondents: section one includes 10 questions on the basic knowledge of the oral health practices and purposes of maintaining oral health. These questions are in the form of multiple-choice questions and respondents were asked to select the correct choice. Each correct answer is given a “one” score while wrong and don’t know choices given a “zero” score. Section two includes seven questions to determine the attitudes of respondents towards oral health. This section evaluated the attitudes of the respondents for the maintenance of the oral health. The questions of this section are yes/no type questions. For the scoring, yes answer is given score “one,” except for the questions with marked as (N), for which score “one” is given for the answer no. The last section of the questionnaire includes 13 items which cover the basic behavior or measures taken by the participants towards the maintenance of the oral health. Out of 13 questions, some are of multiple-choice questions and each correct answer is given score “one.” In this section, there are also some yes/no answer type questions for which yes answer is given score “one,” except for the questions with marked as (N) are given score “one” for the answer no. The validity and reliability of translated version were tested in a pilot study of 35 participants. Cronbach’s alpha was calculated as 0.91.

### Analysis

Data were analyzed using the statistical analysis software SPSS version 20. Descriptive statistics were calculated and mean scores, standard deviation, and frequency distribution were obtained. Independent T-test, correlation coefficients and analysis of variance (ANOVA) were used for data analysis. A *P* of < 0.05 was considered statistically significant.

## Results

A total of 387 respondents out of 450 included samples completed surveys (response rate = 0.84%), comprising 211 (55%) females and 176 (45%) males. The mean age of participants was 23.4 ± 3.9. Demographic characteristics are presented in Table 1.

**Table 1:** Demographic characteristics of participants

Variable	N	%
*Gender*		
Female	211	55
Male	176	45
*Marital status*		
Single	351	91
Married	36	9
*Years of study*		
1st year	82	21
2nd year	79	20
3rd year	119	31
4th year	107	28
*Complementary insurance coverage*		
Yes	84	22
No	303	78
*Household income (monthly)*		
< 20 mR*	32	8
20–40 mR	69	18
40–60 mR	128	33
> 60 mR	158	41

*Mr: Million Rials

Descriptive results of participants’ knowledge, attitudes and behaviors of oral health and their attitudes towards online oral health information are presented in Tables 2 and 3

**Table 2:** Mean scores of knowledge, attitudes and practices of oral health

Variable (range of scores)	Mean ± SD
Knowledge (0–10)	8.89 ± 1.92
Attitudes (0–7)	5.31 ± 1.23
Practices (0–13)	10.27 ± 2.13
KAP (0–30)	23.56 ± 3.18

**Table 3:** Mean Scores of Online health information seeking experience of the participants

Item	Mean ± SD (1–5)
*eHIQ-Part 1*	
Attitudes towards online health information	3.02 ± 0.69
Attitudes towards sharing health experiences online	3.71 ± 1.02
*eHIQ-Part 2*	
Confidence and Identification	2.38 ± 0.96
Information and Presentation	2.98 ± 0.87
Understanding and Motivation	3.79 ± 0.81
eHIQ (total)	3.34 ± 0.83

^*^the mean of eHIQ-Part 1was more than average (*p* < 0.05)

As presented in Table 2, participants had a good knowledge, attitudes and practices of oral health. The results of z-score for all the KAP dimensions were significantly higher than an average (*p* < 0.05) that categorized as a good level of knowledge, attitude and practice. The between-groups differences tests showed that oral health knowledge and attitudes were significantly different between "gender" and "years of study" groups but the differences of oral health practices were significant only based on the "years of study". Results of the Post hoc analysis have indicated the statistical significance among the students in year 1 and 3 and the students in year 1 and four in the dimensions of knowledge, attitude and practice respectively.

Table 3 shows the descriptive statistics of e-Health experience. Participants had moderate scores regarding all sub-scales of eHIQ-Parts 1 and 2. According to the results of z-score and considering 3 as a mean of each dimension (range of answerers between 1 and 5) attitudes of the participants towards online health information and attitudes towards sharing health experiences online indicate an average level (*p* < 0.05) while the participants’ confidence and identification, information and presentation and understanding and motivation were statistically less that average (comparing with 3 as a mean of each dimension) (*p* > 0.05).

Table 4 explores the correlations between the e-HIQ scores and oral health KAP scores of participants. Based on the findings presented in this table, eHIQ has statistical correlations with all the knowledge, attitudes and practices of participants' oral health. The same correlations were seen between e-HIQ subscales and oral health KAP. Also, a complementary analysis showed that better oral health knowledge improves attitudes and practices of oral health.

**Table 4:** Correlations of online oral health information seeking subscales with oral Health KAP

	Knowledge of oral health		Attitudes of oral health		Practices of oral health	
	r	*P*	r	*P*	r	*P*
Attitudes towards online health information	0.43	.04^*^	0.68	.01^*^	0.52	.03^*^
Attitudes towards sharing health experiences online	0.27	.19	0.21	.21	0.35	.11
Confidence and Identification	0.61	.02^*^	0.51	.04^*^	0.49	.02^*^
Information and Presentation	0.54	.00^*^	0.25	.14	0.22	.09
Understanding and Motivation	0.68	.01^*^	0.58	.03^*^	0.61	.03^*^
eHIQ (total)	0.43	.02^*^	0.41	.05^*^	0.62	.04^*^
Knowledge of oral health	–	–	0.74	.00^*^	0.69	.03^*^

^*^significant at *P* < 0.05

## Discussion

This study aimed to assess the correlations between online oral health information seeking experience with the oral health KAP. Findings showed a positive correlation demonstrating that online information seeking affects the knowledge, attitudes and practices of oral health which, subsequently, affect the oral health status.

Results of this study also indicate the average scores obtained by participants in two sub-scales of eHIQ-Part 1 named "Attitudes towards online health information" and "Attitudes towards sharing health experiences online". According to these findings, participants are using the internet in their oral health-related decisions moderately and they thought that the internet could be moderately useful to help people in their oral health-related decision making. Also, they thought that the internet is a good channel to share oral health experiences and to seek people with the same problems. The moderate score of participants regarding "confidence and identification" suggests that they have not a sense of solidarity with other internet users in their information seeking journey; The internet does not give them a sense of confidence to share their health issues with others and they thought that online search is not very helpful for them to better manage their oral health-related conditions. Therefore, they didn’t value highly online health information. The moderate scores of participants regarding the last two subscales of eHIQ-Part 2 including “information and presentation” and “understanding and motivation”, show that the information provided by health websites are moderately presented good but they highly understandable for the participants and encourage and motivate them to play an active role in their health promotion. In this regard, results of Valizadeh-Haghi and Rahmatizadeh (2018) showed a high level of eHealth literacy among the dental patients referring to the private clinics in Tehran, Iran. According to the authors, this condition can help better decision making and more serious information seeking behavior by the patients [13]. Although these differences can be justified by the variety of the study samples and context, it can also emphasize the relationship among the knowledge and literacy and oral health information seeking behavior.

At the same time, other similar results applying eHIQ instrument on the participants over age 18 in UK indicate good psychometric properties in the eHIQ-Part 1 (11 items) and the eHIQ-Part 2 (26 items) [11]. These results that are in line with ours have emphasized that eHIQ among different communities and study population can lead to more information and future windows in providing online health information.

Other present findings regarding oral health KAP showed that participants have relatively good and upper average knowledge, attitudes and practices towards oral health. Kamran et al. (2014) in a similar study evaluated oral hygiene practice, knowledge and attitude among 10–15-year-old school children in Iran. They concluded that oral hygiene habits, oral health awareness and knowledge level among school children are unsatisfactory. Participants had poor oral health behavior, insufficient knowledge, incorrect attitude and practice regarding oral health [14]. However, the study of Rad et al. (2015) which assessed the oral health KAP in 12‑year‑old schoolchildren from the rural and urban areas of five provinces of Iran concluded that 12‑year‑old school children in Iran have good knowledge and positive attitudes, but oral health practices are unsatisfactory [4]. Khamaiseh et al. (2013) in a similar study among secondary school students in the Al-Karak Governorate, Jordan, reported that the mean knowledge score of participants was poor, and concluded that a health education program should be integrated within the school curriculum emphasizing oral health [1]. Smyth et al. (2007), in a study on 12-years school children of Galicia in Spain, reported an important association between oral health knowledge, attitudes and practice. However, their results also showed that attitude is not totally explained by knowledge, so that attitude cannot be understood simply as an intermediate variable in a knowledge-practice causal chain. Specifically, the results indicated that socio-cultural environment modifies the association of knowledge, attitudes and practices [15]. Summarizing and comparing all the above with the present findings may lead to pay more attention by the oral health policymakers to improve the community`s level of health literacy along with the online and digital literacy in order to improve the people health information seeking behavior, get better decisions and achieve more health.

At the same time, despite the high volume of literature on the knowledge, attitudes and practices of oral health, limited studies have considered the internet as a source of oral health information and even an education platform. Bal et al. (2020) investigated the benefits of social media as a tool in dental public health. They explored the advantages and disadvantages of social media and concluded that when social media is used adequately, it has beneficial effects on dental and general health. Though mishandling the power of social media puts a negative impact on people as well as on healthcare providers. Therefore, proper guidelines should be developed about using social media for oral health purposes to get more effective results [16]. Holtzman et al. (2014) in a secondary data analysis investigated the association between oral health literacy and failed appointments in adults attending a university-based general dental clinic in USA. They reported that individuals who use fewer sources of oral health information, a subset of health literacy skills, are more likely to fail to show for dental appointments [17]. Almozainy (2017) in another study assessed the use of social media as a source of information related to dentistry among residents of Saudi Arabia above 18 years of age who could be accessed through social networking sites. They found that about fifty-eight percent of their respondents (n = 1030) search on social media if they face any dental issue and 68.6% of them stated that they usually find the information they were looking for [18]. Haritha et al. (2020) in a survey have examined the oral healthcare information seeking behavior of Indian, Pondicherry university students. This study found that students of Pondicherry University are educated and have full knowledge about general health but have average knowledge about oral health and neglect to take proactive steps to improve their oral health. They also concluded that the main reason for all the problems of oral health is the lack of awareness for oral health information (OHI) [19]. All these findings along with the present results can open the new window for policymakers to enhance modern ways of information seeking among the community. In this regard a particular attention should be paid to those groups the same as the university students as their behavior can be easily influenced on their families and as the medical student on their clients and the whole community. At the same time, making oral health information available and accessible to the public is an important cornerstone of attempts aimed to improve public oral health KAP. Considering the significant growth of internet use as a source of health information, it could be considered as a low cost, easily accessible way to communicate oral health information to the public. Also, the internet could be used as a platform to deliver oral health interventions. However, it should not be forgotten that the internet has some disadvantages besides its many benefits. In health-related uses, some of the most important disadvantages include the dissemination of misinformation that can lead to misunderstanding and be misleading. Also, the disparities of access to the internet between different population groups is an important issue that can exacerbate health disparities. Therefore, different aspects of internet usage for health-related purposes need to be studied further.

Considering all the above and regarding the similar evidence from different countries including India [20–23], Saudi Arabia [24, 25], Egypt [26], Turkey [27], Pakistan [17], China [16, 18] have indicated that in spite of differences among the level of knowledge, attitude and practice in the area of oral and dental health and despite the vast variety in the mechanism, method and rate of oral health information seeking behavior, effective interventions especially educational ones need to be designed to improve the oral health KAP. So it is recommended to design applied educational interventions using the potentiality of the internet and social media to increase the familiarity of the community with the new methods of seeking information and the pros and cons of applying the internet for this purpose.

## Conclusion

According to the results, the general views of using the internet in relation to health and the consequences of using specific health-related online sources were in a moderate level among the participants. Such results can emphasize the need for more planning, education and empowerment of the population`s health literacy by the health policymakers. It can also shed the light for policymakers to develop the online access infrastructures along with enhancing the online health information seeking culture among the community and the students as well.

## Limitations

Our sample consisted of university students that were studying medical sciences which provided self-reported information. Responses from our sample are likely to have been influenced by the socio-cultural context of the students’ background. Therefore, generalization of findings to the wider Iranian population may be inappropriate.

## Data Availability

The datasets generated and/or analyzed during the current study are not publicly available considering that we have not required consents to publish this data, but are available from the corresponding author on reasonable request.
